# fNIRS and behavioral measures of inhibition are correlated with different aspects of impulsivity

**DOI:** 10.1192/j.eurpsy.2025.1679

**Published:** 2025-08-26

**Authors:** A. D. S. Afonso Junior, W. Machado-Pinheiro, A. G. Seabra, M. A. Munoz, L. R. R. Carreiro

**Affiliations:** 1 Universidade Presbiteriana Mackenzie, São Paulo; 2 Univeridade Federal Fluminense, Rio das Ostras, Brazil; 3 Universidad de Granada, Granada, Spain

## Abstract

**Introduction:**

Impulsivity implies difficulties in control, leading to non-premeditated actions, and troubles resisting distraction and remaining focused on a goal. It is a transdiagnostic construct that is associated with several disorders, including Attentional Deficit and Hyperactivity Disorder (ADHD), Borderline Personality Disorder and Obsessive-Compulsive Disorder (OCD). It is possible to divide impulsivity between impulsive actions, associated with the ability to inhibit actions, and choice impulsivity, that involves decision-making. It is also common to differentiate between trait impulsivity, that involves more stable characteristics, and state impulsivity, in which the impulsive behavior is more transient.

**Objectives:**

To investigate the association between action impulsivity as a trait (using self-report measures) and as state (using different level of analysis in a computerized task, i.e., behavioral and neuroimaging measures).

**Methods:**

52 university students (mean age = 21.4; standard deviation = 3.33) filled the BIS-11 self-report questionnaires and completed a Stroop-matching/stop-signal task while they had their behavioral and hemodynamic brain activity collected using Functional Near- Infrared Spectroscopy (fNIRS). The Stroop-matching/stop-signal task had three conditions varying the inhibitory demands: Congruent/Unrelated; Incongruent/Unrelated and Incongruent/Related. Spearman correlations were performed between scores of the BIS-11 subscales (Attentional, Motor and Non-planning impulsivity) and the reaction time (RT) and hemodynamic responses (β) of the Stroop-matching/stop-signal task. Alpha level = 0.05.

**Results:**

RTs in all conditions of the task were positively correlated with Motor Impulsivity scores (Congruent/Unrelated, rho = .31, p = .025; Incongruent/Unrelated, rho = .27, p = .049; Incongruent/Related, rho = .38, p = .005). Brain activity in the left temporoparietal region was positively correlated with Attention Impulsivity scores (rho = .29, p = .033).

**Conclusions:**

Motor and attentional aspects of trait impulsivity can be differently correlated with behavioral and neurophysiological measures of state impulsivity. In this study, motor impulsivity was correlated with more peripherical measures of inhibition (reaction times) while attentional impulsivity was correlated with activity in temporoparietal regions commonly associated with inhibition of distractive stimuli. Greater levels of motor and attentional impulsivity were associated with slower responses and greater brain activity, respectively.

**Disclosure of Interest:**

None Declared

